# Unexpected arterial wall and cellular inflammation in patients with rheumatoid arthritis in remission using biological therapy: a cross-sectional study

**DOI:** 10.1186/s13075-016-1008-z

**Published:** 2016-05-21

**Authors:** Sophie J. Bernelot Moens, Fleur M. van der Valk, Aart C. Strang, Jeffrey Kroon, Loek P. Smits, Eva L. Kneepkens, Hein J. Verberne, Jaap D. van Buul, Michael T. Nurmohamed, Erik S. G. Stroes

**Affiliations:** Department of Vascular Medicine, Academic Medical Center, Room F4-211, PO Box 22660, Amsterdam, 1100 DD The Netherlands; Department of Molecular Cell Biology, Sanquin Research and Landsteiner Laboratory, Academic Medical Center, Amsterdam, The Netherlands; Departments of Rheumatology Reade, Amsterdam Rheumatology and Immunology Center, VU University Medical Center, Amsterdam, The Netherlands; Department of Nuclear Medicine, Academic Medical Center, Amsterdam, The Netherlands

**Keywords:** Rheumatoid arthritis, Cardiovascular disease, Inflammation, Imaging

## Abstract

**Background:**

Increasing numbers of patients (up to 40 %) with rheumatoid arthritis (RA) achieve remission, yet it remains to be elucidated whether this also normalizes their cardiovascular risk. Short-term treatment with TNF inhibitors lowers arterial wall inflammation, but not to levels of healthy controls. We investigated whether RA patients in long-term remission are characterized by normalized inflammatory activity of the arterial wall and if this is dependent on type of medication used (TNF-inhibitor versus nonbiological disease-modifying antirheumatic drugs (DMARDs)).

**Methods:**

Arterial wall inflammation, bone marrow and splenic activity (index of progenitor cell activity) was assessed with ^18^F-fluorodeoxyglucose (^18^F-FDG) positron emission tomography/computed tomography (PET/CT) in RA patients in remission (disease activity score (DAS28) <2.6 for >6 months) and healthy controls. We performed ex vivo characterization of monocytes using flow cytometry and a transendothelial migration assay.

**Results:**

Overall, arterial wall inflammation was comparable in RA patients (n = 23) in long-term remission and controls (n = 17). However, RA subjects using current anti-TNF therapy (n = 13, disease activity score 1.98[1.8–2.2]) have an almost 1.2-fold higher ^18^F-FDG uptake in the arterial wall compared to those using DMARDs (but with previous anti-TNF therapy) (n = 10, disease activity score 2.24[1.3–2.5]), which seemed to be predominantly explained by longer duration of their rheumatic disease in a multivariate linear regression analysis. This coincided with increased expression of pro-adhesive (CCR2) and migratory (CD11c, CD18) surface markers on monocytes and a concomitant increased migratory capacity. Finally, we found increased activity in bone marrow and spleen in RA patients using anti-TNF therapy compared to those with DMARDs and controls.

**Conclusions:**

A subset of patients with RA in clinical remission have activated monocytes and increased inflammation in the arterial wall, despite the use of potent TNF blocking therapies. In these subjects, RA disease duration was the most important contributor to the level of arterial wall inflammation. This increased inflammatory state implies higher cardiovascular risk in these patients, who thus may require more stringent CV risk management.

**Electronic supplementary material:**

The online version of this article (doi:10.1186/s13075-016-1008-z) contains supplementary material, which is available to authorized users.

## Background

Patients with rheumatoid arthritis (RA) are characterized by an approximately twofold increase in cardiovascular disease (CVD) risk [1], which cannot be solely attributed to traditional CVD risk factors [2]. Thus, attention has shifted toward a direct pathogenic role of the systemic inflammatory state in RA. Although up to 40 % of RA patients will reach clinical (articular) remission following treatment [3], it remains to be established whether the increased CVD risk also disappears. Treatment with methotrexate (MTX) in RA patients resulted in a marked reduction in the inflammatory activity, in conjunction with a 21 % lower CVD event rate [4, 5]; yet mortality remains clearly increased compared to the general population [6, 7]. Considering the potent anti-inflammatory effects of immune-modulating biological therapies [8, 9], and in view of the central role of tumor necrosis factor (TNF) in atherogenesis [10], the introduction of TNF inhibitors holds the promise of further reducing this residual CVD burden, although definite outcomes are at present still controversial [11–13].

Supporting its potential role in reducing CVD, anti-TNF treatment was shown to have favorable effects on the arterial wall, with beneficial impact on intima media thickness (IMT) progression as well as arterial wall stiffness [14]. Short-term (8 weeks) TNF inhibition also significantly reduced arterial wall inflammation in patients with active RA, although it failed to completely normalize arterial inflammation to levels observed in control subjects [15]. Whether arterial inflammation can be further reduced during prolonged remission, with or without anti-TNF treatment, remains to be established.

In analogy to the central role of activated monocytes regulating synovial inflammation in RA [16], recent data substantiated a quite similar role for circulating innate immune cells driving arterial wall inflammation in atherosclerotic disease [17]. In experimental atherosclerosis models following an acute coronary event (ACS), increased mobilization of myelopoietic precursors from the bone marrow elicits inflammation of systemic atherosclerotic lesions, mediated by increased influx of these newly formed inflammatory monocytes [18]. However, whether RA-associated cell mobilization/activation contributes to arterial wall inflammation remains to be established.

In the present study, we assessed arterial wall inflammation in RA patients who were in stable clinical (articular) remission. Considering the above-mentioned direct role for TNF in atherosclerosis, we also evaluated the presence of potential drug-specific effects by categorizing RA subjects into those with either stable remission with anti-TNF therapy or stable remission without anti-TNF therapy (but with disease-modifying antirheumatic drugs (DMARDs). Moreover, we assessed monocyte phenotype and function, as well as bone marrow and splenic ‘metabolic’ activity (an index of progenitor cell activity) using ^18^F-fluorodeoxyglucose (^18^F-FDG) positron emission tomography (PET) with computed tomography (CT).

## Methods

### Study population

We performed a controlled cross-sectional cohort study in subjects with an established diagnosis of RA (based on the ACR/EULAR classification [19]), in remission (defined by disease activity score in 28 joints (DAS28) below 2.6 [20]) for more than 6 months. RA subjects were compared to healthy controls, matched for age and sex. Because of ethical constraints concerning radiation exposure, for the imaging studies, healthy controls were selected from a contemporaneous study using identical imaging protocols and performed on the same scanner. For the ex vivo studies, healthy controls (matched for age and sex) were recruited through advertisement. General exclusion criteria were medical history of CVD and the presence of diabetes. To enable comparison of different treatments, while minimizing confounding by indication, all patients were selected based on previous use of anti-TNF therapy. For patients currently on DMARDs, criteria for discontinuation of TNF inhibitors were: at least 6 months of treatment with stable use of concomitant DMARDs, remission based on DAS28 for at least 6 months. The study protocol was approved by the Institutional Review Board of the Academic Medical Center in Amsterdam. Written informed consent was obtained from each participant.

### Baseline data collection

Fasting basal lipid levels, leukocyte count and differentiation, erythrocyte sedimentation rate (ESR) and C-reactive protein (CRP) were determined using standard laboratory procedures. Physical examination, including blood pressure was performed and medical and family history was recorded. In the RA subjects, disease severity was recorded at the time of visit using DAS28-ESR, combining swelling and tenderness in 28 joints, general wellbeing of the patient (visual analogue scale) and ESR levels [21]. Date of disease onset, anti-cyclic citrullinated peptide (anti-CCP), rheumatoid factor (RF) positivity and medication use were extracted from medical history.

### ^18^F-FDG PET/CT imaging

Fasting subjects underwent ^18^F-FDG PET/CT imaging on a PET/CT scanner (Philips, Best, the Netherlands) as previously described [22]. Imaging was initiated, 90 minutes post ^18^F-FDG infusion (250 MBq, 6.8 mCi), with a low-dose, noncontrast-enhanced CT scan for attenuation correction and anatomic co-registration, followed by PET imaging of the thoracic and splenic region.

Images were analyzed with dedicated software (OsiriX, Geneva, Switzerland; http://www.osirix-viewer.com). ^18^F-FDG uptake was assessed in the ascending aorta, bone marrow and spleen. In each artery five regions of interest (ROIs) were drawn. Maximum standardized uptake values (SUV_max_) were averaged for each artery, and divided by the average venous background activity (SUV_mean_) to obtain the target-to-background ratio (TBR_max_). Three consecutive segments with the highest uptake were used to determine the most diseased segment (MDS) [22]. The SUV_max_ values in the bone marrow and spleen were determined as follows; bone marrow activity was calculated as the average SUV_max_ of all imaged vertebrae, and splenic SUV_max_ was obtained as an average of the SUV_max_ obtained in axial, sagittal, and coronal planes, as previously described [23].

### Ex vivo monocyte studies

#### Flow cytometry

Red blood cells were lysed with BD FSCSTM-lysis solution (BD Biosciences, San Diego, CA, USA). Peripheral blood mononuclear cell (PBMCs) were incubated with fluorchrome-labeled antibodies (Additional file 1: Table S1) for 15 minutes and washed with saline. Samples were analyzed using an FACSCalibur (Becton Dickinson, Franklin Lakes, NJ, USA). Delta median fluorescence intensity (MFI), determined with FlowJo software (version 5.4+; Tree Star, Ashland, OR, USA), was calculated by subtracting isotype MFI from MFI of the marker in the corresponding color.

### Transendothelial migration

A transendothelial migration (TEM) assay was performed as described previously [24]. Briefly, after overnight stimulation with TNF-α (10 ng/ml), monocytes were added to primary human arterial endothelial cells (HAEC, Lonza, Baltimore, MD, USA), at a concentration of 1*10^6^ cells/ml for 30 minutes at 37 °C, 5 % CO2 and then fixed with 3.7 % formaldehyde (Sigma-Aldrich, Zwijndrecht, the Netherlands). Images were recorded with a Zeiss Axiovert 200 microscope (Plan-apochromat 10x/0.45 M27 Zeiss-objective; Carl Zeiss Inc., Jena, Germany). Transmigrated monocytes were distinguished from adhered monocytes by their transitions from bright to dark morphology and quantified using the cell counter plugin (http://rsbweb.nih.gov/ij/plugins/cell-counter.html) in the Image-J software (http://rsb.info.nih.gov/nih-image/).

### Statistical analysis

Data were analyzed using Prism version 5.0 (GraphPad Software, La Jolla, CA, USA) and SPSS version 21.0 (IBM Corp,. Armonk, NY, USA). Data are presented as mean ± standard deviation (SD), for normally distributed data or medians with interquartile range [IQR], for nonnormally distributed data unless stated otherwise. Depending on distribution all comparisons of subgroups were performed using Student’s *t* test or Mann-Whitney *U* test, and chi-square tests for categorical variables. Differences in TBR between the groups were assessed with a univariate analysis of covariance (ANCOVA) to account for known cardiovascular risk factors: age, gender and smoking status, and any value that differed at baseline. Univariate and multivariate linear regression (with body mass index (BMI), systolic blood pressure, current smoking and one additional explanatory parameter as covariates) was performed to explore the correlation between RA-specific characteristics and arterial wall inflammation (TBR_max_). A *p* value of <0.05 was considered statistically significant.

## Results

### Baseline characteristics

For the PET/CT study, 23 consecutive RA patients in clinical remission for at least 6 months were included at the outpatient clinics of the Academic Medical Center (AMC) and the Amsterdam Rheumatology and Immunology Center, and compared to 17 age- and sex-matched healthy controls. RA subjects had an unfavorable CV risk profile with more smokers, higher blood pressure and higher BMI. Although CRP levels were generally low, they were higher in RA subjects compared to controls (RA: 18[13–30] mg/L vs control: 13[7–16] mg/L, *p* = 0.022) (Table 1).

**Table 1:** Clinical characteristics of control subjects and RA patients

	Control (n = 17)	RA (n = 23)	*p* value	RA, DMARD (n = 10)	RA, anti-TNF (n = 13)	*p* value
Sex, male/female	7/10	8/15	0.680	3/7	5/8	0.637
Age, years	58 ± 13	58 ± 9	0.894	55 ± 11	60 ± 8	0.193
Body mass index, kg/m^2^	22 ± 2	26 ± 6	0.005	25 ± 6	26 ± 4	0.368
Smoking, yes/no	0/17	8/23	0.022	3/7	4/13	0.708
SBP, mmHg	127 ± 13	143 ± 23	0.014	131 ± 22	151 ± 20	0.028
DPB, mmHg	78 ± 8	87 ± 15	0.036	83 ± 15	90 ± 14^†^	0.270
Total cholesterol, mmol/L	5.4 ± 0.9	5.4 ± 0.9	1.000	5.3 ± 0.9	5.4 ± 0.9	0.613
LDL cholesterol, mmol/L	3.3 ± 0.9	3.6 ± 1.1	0.396	3.4 ± 0.8	3.7 ± 1.3	0.284
HDL cholesterol, mmol/L	1.7 ± 0.4	1.5 ± 0.5	0.135	1.6 ± 0.5	1.4 ± 0.4	0.152
Triglycerides, mmol/L	0.66[0.5–1.0]	1.2[0.9–1.8]	0.001	1.1[0.9–1.4]	1.3[0.9–2.1]	0.393
CRP, mg/L	13[7–16]	18[13–30]	0.022	16[12–48]	19[14–30]	0.888

Values are n (%), mean ± SD or median [IQR,] for skewed data

*RA* rheumatoid arthritis, *DMARD* disease-modifying antirheumatic drug, *TNF* tumor necrosis factor, *SBP* systolic blood pressure, *DBP* diastolic blood pressure, *LDL* low-density lipoprotein, *HDL* high-density lipoprotein, *CRP* C-reactive protein

Within the RA subjects, ten patients were currently using nonbiological DMARDs, after they successfully discontinued their anti-TNF therapy for more than 6 months, whereas 13 patients used anti-TNF therapy. Nine out of the 13 patients on anti-TNF treatment had a previous attempt to discontinue which failed, reaching remission again only upon re-introduction. Age, BMI and baseline lipid levels were comparable between groups. Systolic blood pressure was higher in patients using TNF inhibition (RA, DMARD: 131 ± 22; RA, TNF inhibition; 151 ± 20, *p* <0.05 versus all other groups) (Table 1). DAS28, CRP, ESR as well as percentage seropositive RA were equally distributed between the two groups, but disease duration and duration until first start of TNF inhibition was significantly longer in the RA, TNF-inhibition group. RA-specific parameters and type of RA medication, as well as concomitant cardiovascular medication are listed in Table 2.

**Table 2:** Baseline – RA-specific characteristics

	RA, DMARD (n = 10)	RA, anti-TNF (n = 13)	p value
ESR, mm/H	9 [7–21]	6 [6–17]	0.689
Disease activity score (DAS28)	2.24 [1.3–2.5]	1.98 [1.8–2.2]	1.000
Anti-CCP positive (%)	7 (70)	8 (61)	0.673
Rheumatoid factor positive (%)	8 (80)	7 (54)	0.192
Disease duration, years	7.8 [4.0–10.2]	13.3 [9.3–20.2]	0.012
Anti-TNF	n/a		n/a
- Adalimumab		10	
- Certolizumab		1	
- Etanercept		1	
Disease duration until start anti-TNF, years	1.2 [1.0–1.7]	5.6 [4.3–9.1]	0.001
Anti-TNF withdrawal attempts, yes/no	10/0	9/4	n/a
DMARD			n/a
- Methotrexate	9	11	
- Sulfasalazine	1		
Other Rx			
- Statin	1	1	0.848
- Antihypertensive	5	5	0.580

Values are n (%), mean ± SD or median [IQR,] for skewed data

*RA* rheumatoid activity, *DMARD* disease-modifying antirheumatic drug, *TNF* tumor necrosis factor, *ESR* erythrocyte sedimentation rate, *DAS28* disease activity score based on 28 joints, *CCP* cyclic citrullinated peptide

### ^18^F-FDG PET/CT

**Fig. 1:**
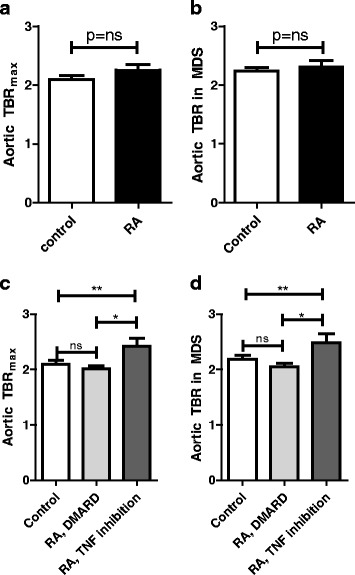
Increased arterial TBR in RA patients on anti-TNF therapy. Quantification of the ^18^F-FDG uptake as the maximum target-to-background ratio (TBR) (**a**, **c**) and TBR in the most diseased section (MDS) (**b**, **d**) in the ascending aorta revealed that RA subjects in stable remission have levels of arterial wall inflammation comparable to healthy controls (**a**, **b**). However, we found increased uptake in RA patients with anti-TNF treatment (n = 13) versus RA subjects on DMARDs (n = 10) and controls (n = 17). *p* values are adjusted for age, gender, BMI, current smoking and systolic blood pressure, * *= p < 0.05*, ** *= p < 0.01. DMARD* disease-modifying antirheumatic drug, *MDS* most diseased segment, *RA* rheumatoid arthritis, *TBR* target-to-background ratio*, TNF* tumor necrosis factor

All parameters were adjusted for cardiovascular risk factors which differed between groups at baseline: systolic blood pressure, current smoking and BMI. Overall, RA subjects in stable remission had levels of arterial wall inflammation compared to matched controls (aortic TBR_max_: RA; 2.3 ± 0.5 versus control; 2.1 ± 0.3, adjusted *p* = 0.251. Aortic TBR_mds_: RA; 23 ± 0.5 versus control; 22 ± 0.3 adjusted *p* = 0.331) (Fig. 1a, b).

Surprisingly, upon further assessment of subgroups, ^18^F-FDG uptake was significantly higher in RA subjects using anti-TNF therapy compared with the RA, DMARD group (with comparable disease activity, CRP and ESR levels) and control subjects, (aortic TBR_max_: RA, TNF inhibition 2.42 ± 0.54 *p* = 0.004 versus controls. RA, DMARD; 2.02 ± 0.16, *p* = 0.011 versus RA, TNF inhibition and *p* = 0.107 versus controls. Similar for TBR_mds_) (Fig. 1c, d).

To assess which factors accounted for the difference in TBR, we performed a univariate and multivariate linear regression using CRP, ESR, DAS28 or disease duration and disease duration till start of TNF inhibition as response variables and TBR_max_ as the dependent variable. In the multivariate analyses, each of these predictors was corrected for the cardiovascular risk factors, which differed at baseline: BMI, systolic blood pressure (SBP) and current smoking (of which only smoking correlated to TBR_max_, Additional file 1: Table S2). Disease duration and duration until start of TNF inhibition correlated with arterial wall inflammation (and in line, with biological use, data not shown), which remained significant after correction for potential confounders (Table 3).

**Table 3:** Unadjusted and adjusted linear regression analysis with TBR_max_ as the dependent variable

Characteristic	Unadjusted analyses		Adjusted analyses^*α*^	
	B (95 % CI)	*p* value	B (95 % CI)	*p* value
ESR	0.009(−0.029-0.048)	0.602	0.024(−0.021-0.070)	0.256
CRP	−0.056(−0.272-0.159)	0.584	−0.075(−0.318-0.168)	0.513
DAS28	0.060(−0.623-0.744)	0.850	0.087(−0.638-0.910)	0.831
Disease duration	0.023(−0.003-0.050)	0.084	0.038(0.002-0.074)	0.040
Disease duration until start of TNF inhibition	0.064(0.015-0.113)	0.015	0.081(0.004-0.158)	0.040

Data are unadjusted coefficient (B) with 95 % confidence intervals (CI)

*TBR*_*max*_ maximum target-to-background ratio, *ESR* erythrocyte sedimentation rate, *CRP* C-reactive protein, *DAS28* disease activity score based on 28 joints

^*α*^Adjusted for BMI, systolic blood pressure and current smoking

### Monocyte phenotype and function

**Fig. 2:**
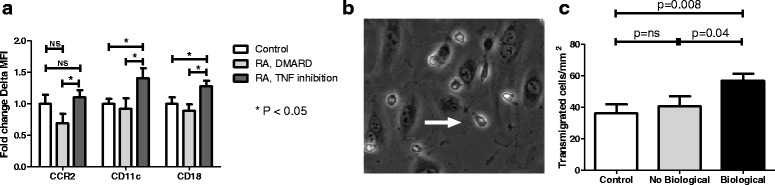
Ex vivo monocyte adhesive and migratory properties. Increased expression (represented as fold change of delta median fluorescent intensity) of monocyte surface markers CCR2, CD11c and CD18 in RA subjects with anti-TNF treatment (n = 20) versus RA patients with DMARDs (n = 16) and controls (n = 20) (**a**). Assessment of transendothelial migratory capacity showed a concomitant increase in number of transmigrated cells (represented as a *white arrow* in (**b**)) (**c**). Data are means + SEM (for each subject, transmigrated cells are calculated of independent counts of five frames of view). * *= p < 0.05*, ** *= p < 0.01. DMARD* disease-modifying antirheumatic drug, *MFI* median fluorescence intensity, *RA* rheumatoid arthritis, *TNF* tumor necrosis factor

We studied phenotype and function of circulating monocytes in RA patient subjects (including additional subjects to the imaging cohort: ten RA patients without anti-TNF therapy who were compared to 13 RA patients with anti-TNF therapy and 20 age- and sex-matched controls. All baseline and RA-specific characteristics were comparable to the imaging study (Additional file 1: Table S3 and Table S4). Analysis of surface markers showed increased expression of CCR2, CD11c and CD18 in RA subjects using anti-TNF therapy versus RA, DMARD and controls (Fig. 2a). Functionally, in a TEM assay, this resulted in a 1.5-fold increase of monocytes, which crossed the endothelial layer (transmigrated cells/mm2: controls 36 ± 24; RA, DMARD 41 ± 19 *p* = ns versus controls; RA, TNF inhibition 57 ± 17, *p* = <0.05 versus all other groups) (Fig. 2b, c).

### Bone marrow and splenic metabolic activity in RA patients

**Fig. 3:**
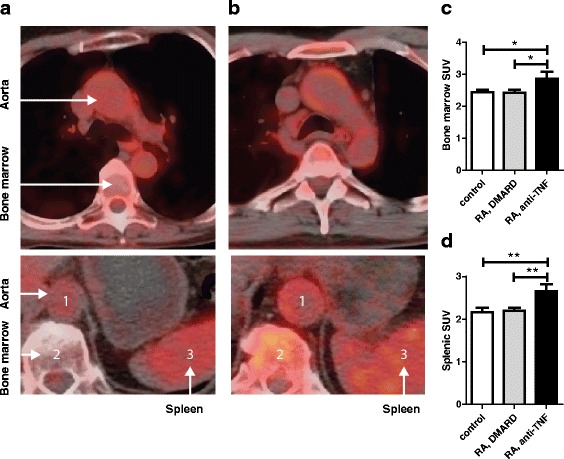
Bone marrow and splenic activity in RA patients. Cross-sectional ^18^F-FDG PET/CT images representing ^18^F-FDG uptake (*red*) in the aorta, bone marrow (*top and bottom*) and spleen (*bottom*) in a RA patient without (**a**) or with anti-TNF treatment (**b**). Quantification of maximum standard uptake values (SUV_max_) showed increased activity in both bone marrow (**c**) and spleen (**d**) of RA patients with continued anti-TNF treatment versus RA patients without anti-TNF treatment and controls. * *= p < 0.05*, ** *= p < 0.01. DMARD* disease-modifying antirheumatic drug, *RA* rheumatoid arthritis, *SUV* standardized uptake values, *TNF* tumor necrosis factor

Finally, we sought to investigate whether the increased presence of promigratory monocytes in RA subjects using anti-TNF therapy would coincide with increased ^18^F-FDG uptake in hematopoietic organs. Indeed, standard uptake values (SUVs) were significantly higher in RA patients using anti-TNF therapy in both bone marrow (BM) and spleen (BM SUV_max_: control 2.44 ± 0.27; RA, DMARD 2.42 ± 0.24, *p* = 0.890 versus controls; RA, TNF inhibition 2.86 ± 0.66, *p* <0.05 versus all other groups. Splenic SUV_max_: control 2.16 ± 0.45; RA, DMARD 2.20 ± 0.21, *p* = 0.817 versus controls; RA, TNF inhibition 2.65 ± 0.48, *p* = 0.01 versus all other groups) (Fig. 3).

## Discussion

In the present study, we show that, overall, RA subjects in long-term clinical remission do not have increased arterial wall inflammation compared to age- and sex-matched controls. However, contrary to the expectations, we observed a markedly increased inflammatory activity in the arterial wall, combined with an activated, promigratory phenotype of circulating monocytes in RA patients using anti-TNF therapy to maintain clinical remission, compared to RA patients using DMARDs, as well as age- and sex-matched healthy control subjects. The concomitantly increased ^18^F-FDG activity in hematopoietic organs (bone marrow and spleen) implies stimulated myelopoiesis contributing to the propagation of the systemic pro-inflammatory state in RA patients in remission using anti-TNF therapy.

Increased ^18^F-FDG uptake in the arterial wall is recognized as an independent predictive marker for future atherothrombotic events [25]. Our study shows that, despite the presence of an adverse CV risk profile, overall, RA subjects in remission had levels of ^18^F-FDG uptake comparable to age- and sex-matched controls. These data support the concept that effective control of inflammatory disease activity diminishes CV risk in RA subjects. However, the FDG uptake in the arterial wall was significantly elevated in RA patients using anti-TNF therapy, reaching levels previously associated with a significant increase in CV risk [25], whereas the signal in patients who used only DMARDs was comparable to that in age- and sex-matched controls. These data imply a persistent inflammatory drive in RA patients in need of continued anti-TNF therapy, despite clinical remission. From a cardiovascular point of view, stopping or tapering anti-TNF in these patients cannot be advocated as one could expect increased vessel wall inflammation, implying an increased CV risk. An obvious challenge for the future is to identify this subset of patients.

As TNF inhibitors were previously shown to effectively lower arterial wall inflammation [15], our findings are most likely explained by inherent differences between treatment groups. Patients with and without anti-TNF therapy were selected retrospectively thus, differences between these groups may reflect heterogeneity in ‘baseline’ disease severity, despite current clinical remission with low disease activity, ESR and CRP levels in both RA groups. To minimize confounding we only included patients who had previously been treated with TNF inhibitors. Moreover, in the group with ongoing anti-TNF therapy, 9 out of 13 patients had attempted to withdraw biological therapy, but failed due to clinical reactivation of articular symptoms (20 out of 20 for the ex vivo studies). Interestingly, Saleem et al. previously showed that short duration of disease symptoms is associated with successful withdrawal of TNF inhibitors [26]. Although we did not assess duration of *active* disease in the present study, total disease duration was longer in subjects who were on current TNF inhibition compared to the group who remained in remission after withdrawal of anti-TNF therapy, and moreover, duration of disease until first start of TNF inhibitors was also significantly longer. Notably, disease duration has previously been identified as an independent risk factor for CVD events [27, 28]. In our study, after correction for multiple confounders, disease duration and time until start of TNF inhibition were the only RA-specific characteristics that significantly correlated to TBR. These observations lend support to the concept that cumulative disease burden in RA may be particularly deleterious [29, 30], illustrating that its contribution to CVD risk cannot be captured in single measurement of disease activity. Eventually, a disease state can be reached where irrevocable changes in the joint [29, 31] or dysregulation of the immune response [26] persists, which may propagate inflammation in distant organs and cells, such as circulating monocytes, known to be key players in the chronic inflammatory state. Whether early use of biological therapies can prevent this [32] and thereby normalizing CVD risk, is a concept that warrants further investigation.

Activation markers of circulating monocytes in plasma predict the CV-event rate in patients [33]. Here, we found a significant increase of CCR2, CD11c and CD18 surface expression on monocytes from RA patients using anti-TNF therapy. Increased expression of CCR2, the receptor for monocyte chemo-attractant protein-1 (MCP-1) promotes chemotaxis [34], and murine studies corroborated that CCR2 is pivotal in the process of plaque formation [35]. The β2 integrin CD11c/CD18 serves as an adhesive ligand by interacting with endothelial VCAM-1, is important in monocyte recruitment and contributes to formation of atherosclerotic plaques [36]. The functional relevance of the increased activation status of circulating monocytes in RA patients using anti-TNF therapy was substantiated by the increased migratory capacity found in the ex vivo TEM assay. In patients with advanced atherosclerotic lesions we previously reported that activated circulating monocytes accumulated more avidly in arterial wall lesions with increased TBR activity on PET/CT [24]. In the present study, our observation in RA subjects on maintenance anti-TNF therapy suggests a similar process, where increased influx of activated plasma monocytes may contribute to increased arterial wall inflammation. Altogether, these data show that RA patients requiring continuation of anti-inflammatory biological treatment may have persistently increased ‘subclinical’ inflammatory activity in both the arterial wall and circulating innate immune cells.

The half-life of circulating monocytes is approximately 1–3 days [37]. This rapid turnover of inflammatory cells highlights the important role for their production sources, the hematopoietic tissues, to continuously supply new immune cells driving (arterial wall) inflammation. In RA patients on anti-TNF therapy, both bone marrow and spleen exhibited increased metabolic activity compared to RA subjects without anti-TNF therapy as well as matched controls. In patients’ post-acute coronary syndrome (ACS), bone marrow and splenic activity were recently found to correlate to arterial wall inflammation as well as to independently predict future CV events [23, 38]. Moreover, activity in BM and spleen correlates to expression of pro-inflammatory markers on circulating leukocytes [18, 23]. The present study reveals increased BM as well as splenic activity in RA patients with clinical quiescent disease under anti-TNF therapy. This elevated activity is associated with both arterial wall inflammation as well as cellular activation, substantiating a multilevel, subclinical, pro-inflammatory phenotype, which has been previously linked to an increased risk for CVD events [39].

### Potential limitations

We conducted a cross-sectional study, in which several limitations need to be considered. First, as aforementioned, confounding by indication may have contributed to our findings, which we tried to minimize regarding RA-related features. However, we cannot account completely for disease severity with the measures provided in this study. Moreover, the design precludes conclusions on disease course and influences of previous medication use. Combined with the small study samples, we thus cannot draw causal conclusions on the observations made in our study. Traditional cardiovascular risk factors are also increased in RA subjects [40], and may be influenced by factors such as disease severity or treatment [11, 41]. In our study, except for systolic blood pressure, which was 20 mmHg higher in RA patients using anti-TNF therapy, traditional CVD risk factors were equally distributed between both RA groups. In line with previous studies [15, 42], we found no correlation between blood pressure and arterial wall inflammation, suggesting that the contribution of systolic blood pressure to arterial wall inflammation in RA subjects is limited. The use of ^18^F-FDG PET/CT for BM and splenic activity has not been fully established and thus interpretation should be done with caution. Particularly concerning BM, distinction between direct involvement in RA and metabolic activity leading to increased production of inflammatory cells cannot be made, as we did not assess synovial activity of the joint affected in RA in the present study. Yet, the vertebrae of the spinal column were used to assess BM activity, an organ not known to be affected in RA.

## Conclusions

We present, for the first time, a study addressing functional arterial wall changes in RA subjects in prolonged stable clinical remission. Overall, subjects in long-term remission had levels of arterial wall inflammation compared to age- and sex-matched controls. However, by using a unique combination of cellular and imaging data, we show that the subset of patients requiring continuation of anti-TNF therapy to maintain remission of their joint disease had increased arterial wall and cellular inflammation compared to those using DMARDs only. In these subjects, RA disease duration was the most important contributor to the level of arterial wall inflammation. This enhanced inflammatory state implies an increased cardiovascular risk in these patients, notwithstanding prolonged clinical remission. In view of this increased CV risk, stringent risk management may be necessary in this group, with perhaps a role for lower treatment thresholds. Finally, the increased hematopoietic activity in these patients also holds a promise for novel therapeutic strategies aimed at attenuating the inflammatory state by targeting immune cell mobilization from the bone marrow.

## Additional file

Additional file 1: Table S1. Antibodies used for flow cytometry. **Table S2.** Univariate linear regression analysis in RA subjects. **Table S3.** Baseline – ex vivo experiments. **Table S4.** Ex vivo baseline – RA-specific characteristics. (PDF 45 kb)

## Abbreviations

^18^F-FDG: ^18^F-fluorodeoxyglucose
ACS: acute coronary syndrome
BM: bone marrow
BMI: body mass index
CCP: cyclic citrullinated peptide
CRP: C-reactive protein
CT: computed tomography
CVD: cardiovascular disease
DAS: disease activity score
DBP: diastolic blood pressure
DMARD: disease-modifying antirheumatic drug
ESR: erythrocyte sedimentation rate
HDL: high-density lipoprotein cholesterol
IMT: intima media thickness
LDL: low-density lipoprotein cholesterol
MDS: most diseased segment
MFI: median fluorescence intensity
MTX: methotrexate
PET: positron emission tomography
RA: Rheumatoid arthritis
RF: rheumatoid factor
ROI: regions of interest
SBP: systolic blood pressure
SUV: standardized uptake values
TBR: target-to-background ratio
TEM: transendothelial migration
TNF: tumor necrosis factor
