# Erratum to: Unexpected arterial wall and cellular inflammation in patients with rheumatoid arthritis in remission using biological therapy: a cross-sectional study

**DOI:** 10.1186/s13075-016-1198-4

**Published:** 2016-12-13

**Authors:** Sophie J. Bernelot Moens, Fleur M. van der Valk, Aart C. Strang, Jeffrey Kroon, Loek P. Smits, Eva L. Kneepkens, Hein J. Verberne, Jaap D. van Buul, Michael T. Nurmohamed, Erik S. G. Stroes

**Affiliations:** 1 Department of Vascular Medicine, Academic Medical Center, Room F4-211, PO Box 22660, Amsterdam, 1100 DD The Netherlands; 2 Department of Molecular Cell Biology, Sanquin Research and Landsteiner Laboratory, Academic Medical Center, Amsterdam, The Netherlands; 3 Departments of Rheumatology Reade, Amsterdam Rheumatology and Immunology Center, VU University Medical Center, Amsterdam, The Netherlands; 4 Department of Nuclear Medicine, Academic Medical Center, Amsterdam, The Netherlands

## Erratum

Unfortunately, after publication of this article [1], it was noticed that the Funding section in the declarations was incomplete. The full correct Funding section can be seen below.
