# Supplementary material for: Unexpected arterial wall and cellular inflammation in patients with rheumatoid arthritis in remission using biological therapy: a cross-sectional study
Source: Arthritis Res Ther. 2016 May 21;18:115. doi: 10.1186/s13075-016-1008-z (PMC4875657; doi:10.1186/s13075-016-1008-z)
Supplement: Additional file 1: Table S1. — Antibodies used for flow cytometry. Table S2. Univariate linear regression analysis in RA subjects. Table S3. Baseline – ex vivo experiments. Table S4. Ex vivo baseline – RA-specific characteristics. (PDF 45 kb) [file 13075_2016_1008_MOESM1_ESM.pdf]

SUPPLEMENTAL MATERIAL

Supplemental Table 1. Antibodies used for flow cytometry

Surface marker	Color	Company
CD14	PE-Cy7	BD
CD16	APC-Cy7	BD
HLA-DR	PerCpCy5.5	BD
CD192 (CCR2)	APC	BD
CD11C	APC	BD
CD18	APC	BD

PE indicates phycoerythrin , Cy; CyChrome, APC; allophycocyanin, HLA; human leukocyte antigen, PerCP; peridinin-chlorophyll-protein.

Supplemental Table 2: Unadjusted linear regression analysis in RA subjects with TBR Max as the dependent variable

**Characteristic**	β (95% CI)	P value
Age	-0.322(-0.039-0.007)	0.165
BMI	0.096(0.385-0.705)	0.705
SBP	-0.064(-0.012-0.009)	0.793
Current Smoking	0.545(-0.066-0.519)	0.014

Data are standardized coefficient (β) with 95% confidence intervals (CI). BMI indicates body mass index, SBP; systolic blood pressure.

Supplemental Table 2: Clinical characteristics– *ex vivo* experiments

	Control (n=20)	RA, no anti-TNF (n=16)	RA, anti-TNF (n=20)
**Sex, male/female**	9/11	3/13	7/13
**Age, years**	56±10	53±10	54±9
**Body mass index, kg/m^2**	26±4	25±6	26±4
**Smoking, yes/no**	2/20	6/16	5/20
**SBP, mm Hg**	131±13	125±20	139±20*
**DPB, mm Hg**	84±10	82±13	84±10
**Total cholesterol, mmol/L**	5.5±1	5.4±0.7	5.0±0.9
**LDL cholesterol, mmol/L**	3.4±0.9	3.3±0.7	2.7±0.9*
**HDL cholesterol, mmol/L**	1.6±0.5	1.7±0,4	1.8±0,6
**Triglycerides, mmol/L**	0.8[0.7-1.1]	0.9[0.8-1.3]	0.8[1.3]
**WBC, 10E9/L**	5.5±0.7	5.5±1.5	5.9±1.6
**- Lymfocytes**	- 1.8±0.5	- 2.8±5.1	- 2.0±0.7
**- Neutrofils**	- 3.0±0.7	- 3.2±1.4	- 3.2±1.1
**- Monocytes**	- 0.5±0.1	-0.5±0.2	- 0.6±0.2

Values are n, mean ± SD *or median [IQR,] for skewed data. SBP indicates systolic blood pressure, SDP; diastolic blood pressure, LDL; low density cholesterol, HDL, high density cholesterol, WBC; white blood cell count. *P<0.05 vs all other groups.*

Supplemental table 3: RA specific characteristics - *ex vivo* experiments

Characteristic	RA, no anti-TNF (n=16)	RA, anti-TNF (n=20)	P-value
**C-reactive protein, mg/L**	1.7[1.1-4.3]	2.0[0.7-3.0]	0.789
**ESR, mm/H**	8[7-18]	6[2-10]	0.042
**Disease activity score (DAS28)**	2.07[1.4-2.4]	1.80[1.3-2.2]	0.432
**Anti-CCP positive (%)**	10 (63)	13 (65)	0.619
**Rheumatoid factor positive (%)**	11 (69)	11 (55)	0.462
**Disease duration, years**	9.1[5.3-14.0]	16.8[11.8-20.3]	0.008
**Anti-TNF**	n/a		n/a
**- Adalimumab**		12	
**- Certolizumab**		1	
**- Etanercept**		7	
**Disease duration until start anti-TNF, years**	1.3[1.0-1.8]	5.3[2.1-9.5]	0.008
**Biological withdrawal attempts, yes/no**	16/0	20/0	n/a
**DMARD**			n/a
**- Methotrexate**	10	17	
**- Sulfasalazine**	1		
**Other Rx**			
**- Statin**	0	1	0.364
**- Anti-hypertensive**	4	6	0.739

Values are n or mean ± SD. *ESR; erythrocyte sedimentation rate, DAS28 indicates disease activity score based on 28 joints, CCP; cyclic citrulline peptide, DMARD; disease modifying antirheumatic drugs. * P<0.05 vs no biological.*
